# Decreased FSP1 expression under chronic hypoxia sensitizes human macrophages towards ferroptosis

**DOI:** 10.1016/j.redox.2026.104406

**Published:** 2026-09-17

**Authors:** Anja Wickert, Anna Schwantes, Yara Shadid, Sofie P. Meyer, Bernhard Brüne, Dominik C. Fuhrmann

**Affiliations:** a Institute of Biochemistry I, Faculty of Medicine, Goethe University Frankfurt, Frankfurt, Germany; b Frankfurt Cancer Institute, Goethe University Frankfurt, Germany

**Keywords:** NRF2, Lipid peroxidation, THP-1, AIFM2

## Abstract

Ferroptosis, an iron-dependent cell death, emerged as a new therapeutic approach to treat diseases such as cancer or diabetes. Several pathological conditions are characterized by the appearance of chronic hypoxia, which is known to attract immune cells, especially monocytes and macrophages. Therefore, we investigated how chronic hypoxia affects ferroptosis in primary human macrophages and THP-1 cells. Chronic but not acute hypoxia sensitized cells towards ferroptosis, in line with elevated cellular as well as mitochondrial lipid peroxides, and mitochondrial ROS. Time kinetics revealed an earlier increase in lipid peroxidation under chronic hypoxia compared to normoxia, which was followed by mitochondrial lipid peroxidation and mitochondrial ROS. Mechanistically, and in contrast to normoxia and/or acute hypoxia, chronic hypoxia diminished nuclear abundance and activity of the antioxidant transcription factor NRF2. Consequently, NRF2 target gene expression, including ferroptosis suppressor protein 1 (FSP1), decreased. Inhibition or knockdown of FSP1 mimicked the ferroptosis-sensitizing effect of chronic hypoxia. To causatively link decreased NRF2 activity to increased ferroptosis under hypoxia, we inhibited KEAP1, which preserved NRF2 activity, increased FSP1 expression, and decreased ferroptosis. Our data provides novel insights into NRF2 regulation under chronic hypoxia linked to FSP1 expression and ferroptosis sensitivity of primary human macrophages.

## Highlights

• Chronic but not acute hypoxia fosters ferroptosis in human macrophages.
• Chronic hypoxia accelerates RSL3-mediated lipid peroxidation and ROS.
• The NRF2-FSP1 signaling axis is disturbed by chronic hypoxia.
• FSP1 inhibition and knockdown mimics the hypoxic phenotype.
• KEAP1 inhibition protects hypoxic cells from ferroptosis.

## Graphical abstract

**Figure:**
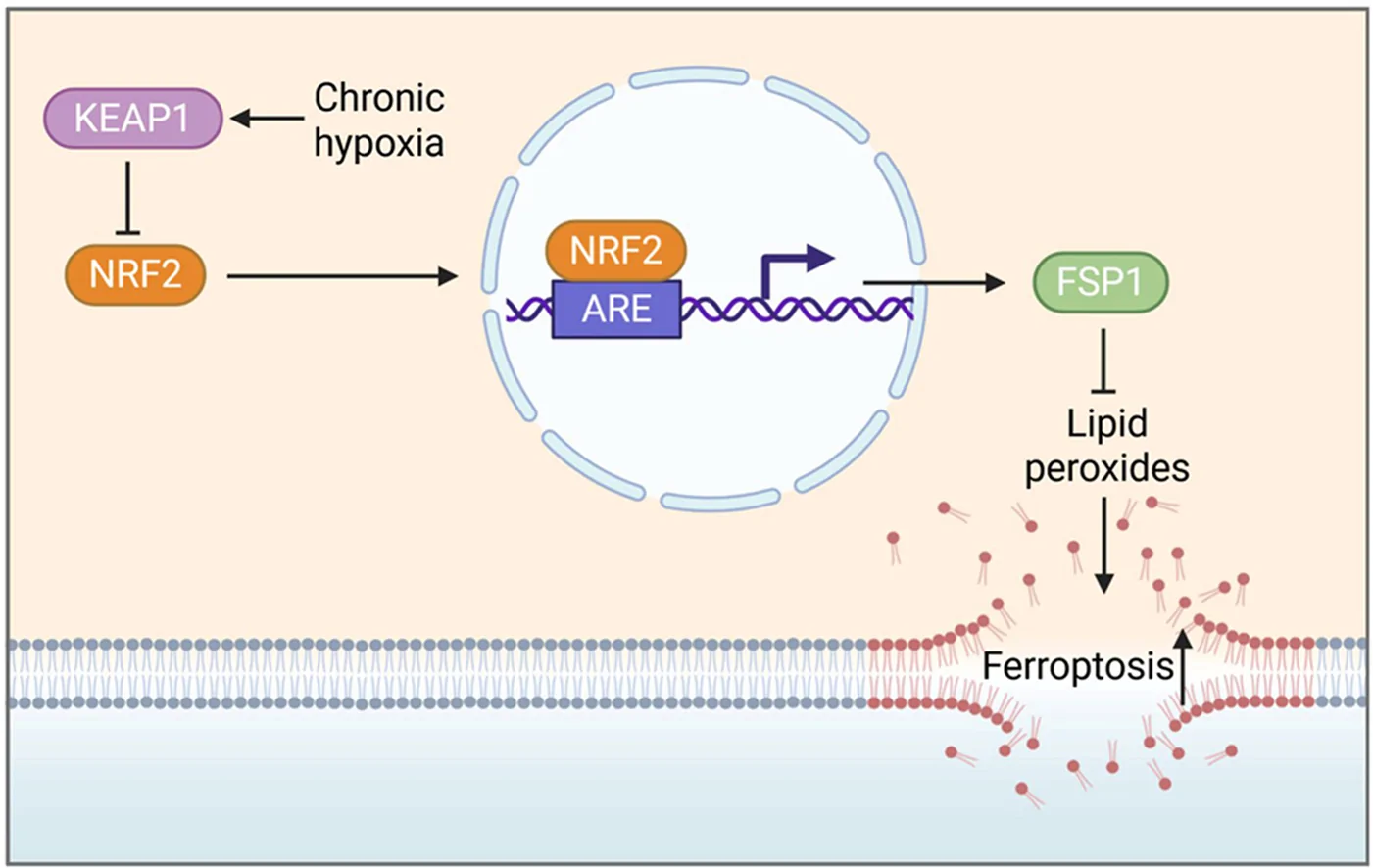

## 1 Introduction

Hypoxia occurs when the need of oxygen exceeds its supply and is associated with physiological conditions, i.e. in the bone marrow or associated with pathological environments found in tumors or inflamed areas [1,2]. Hypoxia is neither defined in terms of severity nor duration and acute versus prolonged or chronic hypoxia often remains undiscriminated. Some attempts by our group and others reached out to distinguish chronic from acute hypoxia [3,4]. Thereby, gene expression analyses revealed an acute, hypoxia-inducible factor 1-mediated response from 8 to 24 h, which decreased from 48 h on. In those studies, chronic hypoxia was defined as a state where gene expression of acute response genes decreased to levels slightly above normoxia and then remained stable for up to 72 h. In addition, under chronic hypoxia genes such as cathepsin B, sorting nexin 5 or H4 clustered histone 1 increased, while activity and abundance of mitochondrial complex I decreased, indicating adaptation of metabolism [[5], [6], [7]].

During hypoxia, oxidative stress plays a pivotal role especially for macrophage polarization as well as executing antimicrobial defense and inflammatory signaling [[8], [9], [10], [11], [12], [13], [14]]. Literature about reactive oxygen species (ROS) production under hypoxia is controversial. Some studies observed decreased mitochondrial ROS production under acute and chronic hypoxia [15,16]. Others report increased ROS production, even though oxygen and its use in oxidative phosphorylation are decreased under hypoxia [[17], [18], [19]]. Another form of oxidative stress are lipid peroxides which are less explored but like ROS are controversially discussed in literature [20,21]. Therefore, balancing oxidative vs. antioxidative pathways is of utmost importance since excessive production of lipid peroxides and ROS damage cells. These forms of oxidative stress promote a type of cell death known as ferroptosis, whose link to hypoxia has not yet been fully elucidated [[22], [23], [24], [25]]. Ferroptosis is an iron-dependent regulated cell death, driven by lipid peroxidation [26]. Lipid peroxidation is fueled by the Fenton reaction where ROS and ferrous ions generate highly toxic hydroxyl radicals resulting in lipid peroxide formation [27]. Upon initiation, lipid peroxidation is a self-propagating process generating new ROS and perpetuating cellular damage [28,29]. To interfere with ferroptosis, protective mechanisms including glutathione peroxidase 4 (GPX4) and/or ferroptosis suppressor protein 1 (FSP1) also known as AIFM2 are in place. GPX4 directly reduces lipid hydroperoxides and inhibition of GPX4 with Ras-selective lethal 3 (RSL3) is used to induce ferroptosis [30], while FSP1 reduces ubiquinone to ubiquinol, which functions as a radical trapping agent [[30], [31], [32]].

FSP1 as well as other antioxidants are regulated by the antioxidant transcription factor nuclear factor erythroid 2-related factor 2 (NRF2) [[33], [34], [35]]. NRF2 acts as important modulator to protect against ROS and therefore ferroptosis. Antioxidant genes are expressed when NRF2 binds to antioxidant response elements. [36]. Kelch-like ECH-associated protein (KEAP1) functions as negative regulator of NRF2 by facilitating polyubiquitination and proteasomal degradation of NRF2 [37,38]. Upon oxidative stress, KEAP1 is oxidized, which prevents its binding to NRF2 thus, resulting in translocation of NRF2 to the nucleus and transcription of antioxidant target genes [[33], [34], [35]].

In this study, we investigate the effect of chronic hypoxia on ferroptosis sensitivity in human macrophages. Chronic hypoxia decreased NRF2 activity and diminished the expression of antioxidant genes including FSP1, thereby increasing lipid peroxidation and ferroptosis.

## 2 Materials and methods

### 2.1 Cell culture of THP-1 cells

THP-1 cells were purchased from ATCC and cultured in RPMI medium (Gibco™, ThermoFisher, Waltham, MA, USA) with 10% FCS and 1% penicillin/streptomycin at 37 °C and 5% CO_2_. For experiments, the cells were counted and seeded in fresh medium.

### 2.2 Isolation of primary human macrophages

For culturing primary human macrophages, peripheral blood mononuclear cells (PBMCs) were isolated from healthy blood samples provided by the blood donor service of the DRK Blutspendedienst Baden-Württemberg-Hessen, Frankfurt, Germany. To separate leukocytes from erythrocytes and plasma, the samples were centrifuged in Leucosep tubes using Biocoll Separation Solution filled up with PBS/EDTA at 440 x g for 35 min and brake set on 2. After centrifugation, the buffy coat between the erythrocytes and plasma was transferred to a fresh tube and washed 3 x with PBS/EDTA (centrifugation at 500 x g, 5 min, room temperature). Then, the pellet was resuspended in serum-free RPMI medium and seeded onto high adherence culture dishes (Advanced TC Surface plates, Greiner BIO-ONE, Frickenhausen, Germany). Subsequently, the samples were incubated at 37 °C for 1 – 2 h before replacing the previous medium with RPMI medium containing 3% human serum and 1% Penicillin/Streptomycin. After 7 days, primary monocytes were differentiated into macrophages. Medium was exchanged every second day with fresh RPMI medium including 3% human serum and 1% penicillin/streptomycin.

### 2.3 Treatments

THP-1 cells and primary human macrophages were treated with 5 μM RSL3 (Cayman chemicals, Ann Arbor, USA) and 1 μM liproxstatin-1 (Cayman chemicals, Ann Arbor, USA). For hypoxic treatments, the cells were cultured in a hypoxia chamber (SCI-tive hypoxia workstation, Baker Ruskinn, Leeds, UK) with 1% O_2_ and 5% CO_2_ at 37 °C. In this work, chronic hypoxia is reached after at least 7 days under hypoxic conditions. Experiments were seeded in fresh hypoxic medium. CDDO-Imidazole (Cayman chemicals, Ann Arbor, USA, CDDO-Im, 25 or 50 nM), iFSP (Cayman chemicals, Ann Arbor, USA,10 μM), erastin (Sigma Aldrich, Darmstadt, Germany, 10 μM), buthionine sulfoximine (Cayman chemicals, Ann Arbor, USA, BSO, 10 or 50 μM), and ES-936 (TargetMol, Boston, USA, 100 or 500 nM) were used at indicated concentrations for the indicated time. Vehicle controls were performed using dimethyl sulfoxide (DMSO).

### 2.4 siRNA transfection

Primary human macrophages were incubated in RPMI without human serum 16 h prior transfection. Cells were transfected with 50 nM ON-TARGET plus siRNA against AIFM2 (L-004443-00-0005), NFE2L2 (L-003755-00-0005) and KEAP1 (L-012453-00-0010) purchased from Horizon Discovery (Cambridge, UK) using HiPerFect transfection reagent (Qiagen, Hilden, Germany).

### 2.5 Viability assays

THP-1 cells and primary human macrophages were seeded in 48-well plates and treated with the respective treatments. After the treatments, viability was determined using CellTiter-Blue® Viability Assay (Promega, Walldorf, Germany). For this, the cells were removed from hypoxia, then incubated with CellTiter-Blue® for 1 h under cell culture conditions. Formation of resorufin as indicator of cell viability was measured by fluorescence (λ_ex_: 560 nm, λ_em_: 590 nm) using Tecan Spark® (Tecan, Männedorf, Switzerland).

### 2.6 ATP release assay

Cell death was determined by measuring extracellular ATP using RealTime-Glo™ Extracellular ATP Assay (Promega, Walldorf, Germany). THP-1 cells were cultured in RPMI medium with 10% FCS and 1% penicillin/streptomycin, buffered with 25 mM HEPES (Carl ROTH, Karlsruhe, Germany). Hypoxic cells were removed from the hypoxia chamber immediately before starting the experiment. Measurements were performed according to the manufacturer's instructions. Luminescence was detected using Tecan Spark®. Samples were mixed for 1 min and measured every 10 min over a period of 17 h.

### 2.7 Seahorse assay

THP- cells were treated for 1 h or 2 h with 5 μM RSL3, centrifuged, and seeded at 5 x 105 cells per well in 180 μL XF RPMI (5 mM glutamine, 2 mM pyruvate, 10 mM glucose) on a XF96 PDL Cell Culture Microplate (Agilent Technologies, Waldborn, Germany). Plates were equilibrated for 30 min in a non-CO2 incubator at 37 °C. Oxygen consumption was measured on a Seahorse XFe 96 extracellular flux analyzer (Agilent). 3 cycles were performed per injection each consisting of 3 min measurement and 3 min mixing. To analyze mitochondrial respiration, 2.5 μM oligomycin, 4 μM carbonyl cyanide m-chlorophenylhydrazone (CCCP), and 500 nM rotenone together with antimycin A were sequentially added to the cells. All chemicals were purchased from Cayman chemicals (Ann Arbor, USA). Data were normalized to cell count and processed using Wave Desktop (Version 2.6.0.31).

### 2.8 Reverse transcription quantitative PCR

RNA was harvested using TRIzol reagent (Invitrogen, Thermo Fisher Scientific, Waltham, MA, USA) followed by a phenol-chloroform extraction. RNA concentrations were determined by UV spectrophotometry at 260 nm using Nanodrop ND-1000 spectrophotometer (Peqlab, Erlangen, Germany). cDNA synthesis was performed using Maxima First Strand cDNA Synthesis Kit for RT-PCR (Thermo Fisher Scientific, Waltham, MA, USA). RNA expression was determined using PowerUp SYBR Green Master Mix (Applied Biosystems, Thermo Fisher Scientific) on QuantStudio 5 PCR Detection System (Applied Biosystems, Thermo Fisher Scientific). Primers for the different targets are listed in Table 1. Measured data was analyzed by the ΔΔct method and normalized to the expression of the housekeeping gene TATA-box-binding protein (TBP).

**Table 1:** List of primers.

Target	Forward	Reverse
TBP	GCATCACTGTTTCTTGGCGT	CGCTGGAACTCGTCTCACTA
FSP1	GACTCCTTCCACCACAATGTGG	CAGCACCATCTGGTTCTTCAGG
GCLC	CAAGCACCCTCGCTTCAGTA	CCGGCTTAGAAGCCCTTGAA
KEAP1	TCCCCAACCGACAACCAAGA	TCCAGGGTGTAGCTGAAGGT
NQO1	TGAAAGGCTGGTTTGAGCGA	GCCTTCTTACTCCGGAAGGG
NRF2	AACTACTCCCAGGTTGCCCA	GACTGGGCTCTCGATGTGAC
SLC7A11	GGTCCATTACCAGCTTTTGTACG	AATGTAGCGTCCAAATGCCAG

### 2.9 Cell lysate and nuclear and cytoplasmic extract preparation

Cells were lysed using lysis buffer containing 10 mM Tris-HCl, 6.65 M urea, 10% (v/v) glycerol, 1% (w/v) SDS, 1x protease inhibitor mixture (Roche, Basel, Switzerland), 1 mM DTT, pH 7.4 and sonicated. Hypoxic cells were lysed under hypoxic conditions. Nuclear and cytosolic fractions were extracted using hypotonic buffer containing 10 mM HEPES pH 7.5, 4 mM NaF, 10 μM Na_2_MoO_4_, 100 μm EDTA, 1x protease inhibitor mixture, 1 mM DTT incubating on ice for 15 min before adding 10% NP40. After centrifugation at 16,000 x g for 30 s, supernatant (cytosolic fraction) was transferred to a fresh reaction tube. The pellet containing nuclei was washed with PBS, lysed in lysis buffer, and sonicated.

### 2.10 Western analysis

For Western analysis, protein concentrations of the cell lysates and fractions were determined using Lowry assay using DC™ Protein Assay Kit (Bio-Rad, Munich, Germany). 60 μg of protein were denatured at 95 °C and loaded on 10 - 15% SDS gels. Gels were blotted using Trans Blot Turbo blotting system (Bio-Rad). Before blocking, membranes were stained using the Revert™ 700 Total Protein Stain kit (Licor, Lincoln, USA). The membranes were blocked in 5% BSA in TBS-T for KEAP1, NQO1 and FSP1 and with 5% milk in TBS-T for NRF2, followed by incubation with the primary antibodies α-actin (1:3000 in 5% milk, Sigma-Aldrich, ACTA1), FSP1 (1:1000 in 5% BSA, Cell Signaling, 24972S), KEAP1 (1:5000 in 5% BSA, ProteinTech, 10503-2-AP), NQO1 (1:500 in 5% BSA, Santa Cruz, sc-32793), NRF2 (1:6000 in 5% milk, ProteinTech, 16396-1-AP) and nucleolin (1:3000 in 5% milk, Santa Cruz, sc-55486). IRDye secondary antibodies (LI-COR Biosciences, Bad Homburg, Germany) were incubated 1:10,000 in 5% milk. Fluorescence signal was detected on an Odyssey scanner (LI-COR Biosciences) and quantified with Image Studio Digits 5.0 (LI-COR Biosciences). The signal was normalized using a lane normalization factor (LNF; intensity of a complete lane divided by the intensity of the lane with the maximal intensity) or the respective fractionation control (cytosol: α-actin; nucleus: nucleolin).

### 2.11 Imaging flow cytometry

Lipid peroxidation (BODIPY C11), mitochondrial lipid peroxidation (mitoCLox), mitochondrial ROS (mitoSOX) and cell death (prodidium iodide, PI) were analyzed by flow cytometric imaging (Amnis ImageStreamX Mk II, Cytek, Amsterdam, Netherlands). For this, 0.5 × 10^6^ cells were seeded and treated with respective treatments. After the treatment, the cells were stained with 5 μM of BODIPY C11 (Invitrogen, Thermo Fisher Scientific), 200 nM of mitoCLox (Lumiprobe, Moscow, Russia) or 500 nM of mitoSOX™ Red (Invitrogen, Thermo Fisher Scientific) in PBS containing 2% FCS for 30 min under cell culture conditions. Hypoxic cells were stained under hypoxic conditions. After staining, cells were washed with PBS and resuspended in PBS containing 2% FCS for measurement. For determining cell death, cells were washed with PBS and stained with 1 μg/mL PI in PBS in the dark on ice for at least 10 min. Afterwards, the samples were measured without removing the staining. From each sample, 2000 cells, which were identified as single cells and in focus, were measured and used for analyses. Pictures were obtained with 40x magnification. Data were analyzed with IDEAS® Image Analysis Software.

### 2.12 Statistics

All experiments were performed at least three times. Each biological replicate contains a control and was normalized accordingly. Statistics were performed with GraphPad Prism 10.5.0. Data are expressed as mean values ± SD. Statistically significant differences were calculated after analysis of variance (ANOVA) or Student's t-test; p ≤ 0.05 was considered significant (*: p ≤ 0.05; **: p ≤ 0.01; ***:p ≤ 0.001; ****: p ≤ 0.0001).

## 3 Results

### 3.1 Chronic hypoxia sensitizes human macrophages towards ferroptosis

**Fig. 1:**
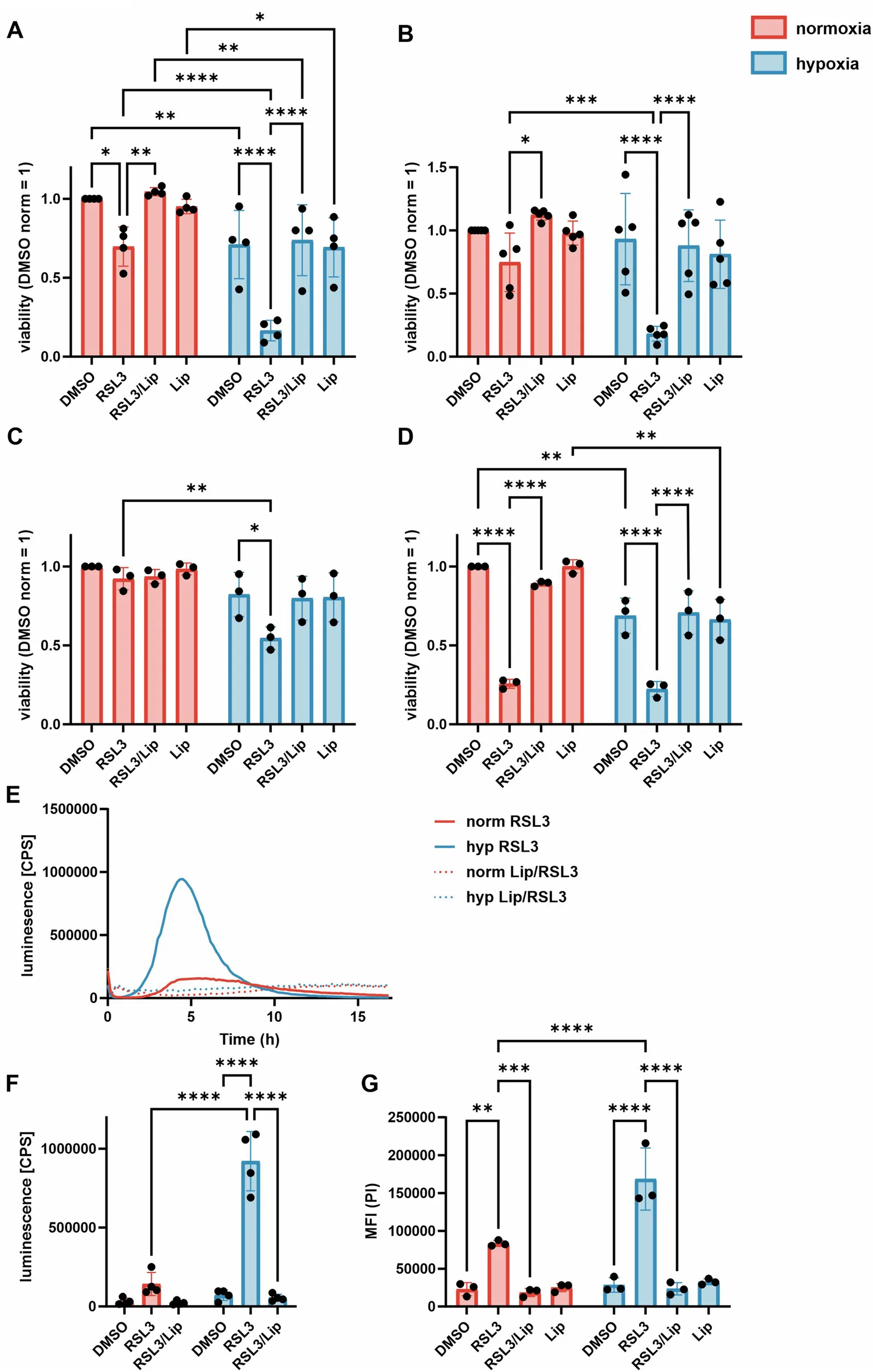
**Chronic hypoxia sensitizes human macrophages towards ferroptosis.** Primary human macrophages were cultured under normoxia or chronic hypoxia and treated with 5 μM RSL3 ± 1 μM liproxstatin-1 (Lip) or DMSO as vehicle control for 6 h (A) and 24 h (B). THP-1 cells were cultured under normoxia and chronic hypoxia and treated with 5 μM RSL3 ± 1 μM Lip or DMSO as vehicle control for 6 h (C) and 20 h (D). Viability was determined by CellTiter-Blue® Viability Assay. (E − F) THP-1 cells were cultured under normoxia and chronic hypoxia and treated with 5 μM RSL3 ± 1 μM Lip or DMSO as vehicle control for in total 17 h. Extracellular ATP was measured using RealTime-Glo™ Extracellular ATP Assay. (E) Luminescence measurements were performed every 10 min and are expressed in counts per second (CPS). (F) Luminescence at 4 h after treatment. (G) THP-1 cells were cultured under normoxia and chronic hypoxia and treated with 5 μM RSL3 ± 1 μM Lip or DMSO as vehicle control for 4 h before propidium iodide (PI) staining. Fluorescence of incorporated PI was measured using imaging flow cytometry. Results are expressed as mean fluorescence intensities (MFI). Data are expressed as mean ± SD. Two-way ANOVA with Tukey's multiple comparisons test was used. p ≤ 0.05 was considered significant (*: p ≤ 0.05; **: p ≤ 0.01; ***: p ≤ 0.001; ****: p ≤ 0.0001).

Chronic hypoxia is a feature of tumors or inflamed tissue and promotes accumulation of macrophages at these sites [10]. Therefore, we questioned how chronic hypoxia affects ferroptosis sensitivity of human macrophages. PBMCs were isolated from human blood, differentiated with serum, cultured under hypoxia for 7 days, followed by treatment with RSL3 to induce ferroptosis. Viability assays revealed increased ferroptosis under chronic hypoxia compared to normoxia after 6 h (Fig. 1A) and 24 h (Fig. 1B) of RSL3 treatment. To verify ferroptotic cell death, we used the lipid peroxidation scavenger liproxstatin-1, which prevented RSL3-induced cell death. As mechanistic investigation using primary human macrophages is challenging, we used the monocytic cell line THP-1 as a model and recapitulated effects observed in primary cells. THP-1 cells were cultured for 7 days under hypoxia and treated with RSL3 to confirm the sensitizing impact of chronic hypoxia (Fig. 1C). Also, in THP-1 cells liproxstatin-1 fully prevented ferroptosis. However, THP-1 cells underwent ferroptosis after a prolonged, 20 h-lasting RSL3-treatment under normoxia, an effect not seen in primary macrophages (Fig. 1D vs. Fig. 1B). We also treated THP-1 cells with erastin or ML162 (Fig. S1A and B). Interestingly, only direct GPX4 inhibition by ML162 recapitulated RSL3 effects. To better understand the kinetics of membrane damage under chronic hypoxia during ferroptosis, we measured ATP release over time (Fig. 1E). ATP release under chronic hypoxia increased faster and was more pronounced compared to normoxia (Fig. 1F) and was completely prevented by liproxstatin-1. Since viability assays as well as ATP release represent metabolic assays, we stained cells with PI for further validation (Fig. 1G). RSL3-treated chronic hypoxic cells accumulated significantly more PI compared to normoxic cells. In a next step, we analyzed mitochondrial integrity by Seahorse measurements. While uncoupling the respiratory chain increased respiration under normoxia and chronic hypoxia to a similar extent compared to the corresponding controls (Fig. S1C), RSL3 decreased mitochondrial respiration already after 1 h of treatment under chronic hypoxia, indicating a pronounced loss of mitochondrial integrity under chronic hypoxia compared to normoxia (Fig. S1D). Conclusively, chronic hypoxia sensitized human macrophages as well as THP-1 cells towards ferroptosis, especially at short RSL3-incubation times.

### 3.2 Chronic hypoxia elevates and accelerates lipid peroxidation and mitochondrial ROS production

**Fig. 2:**
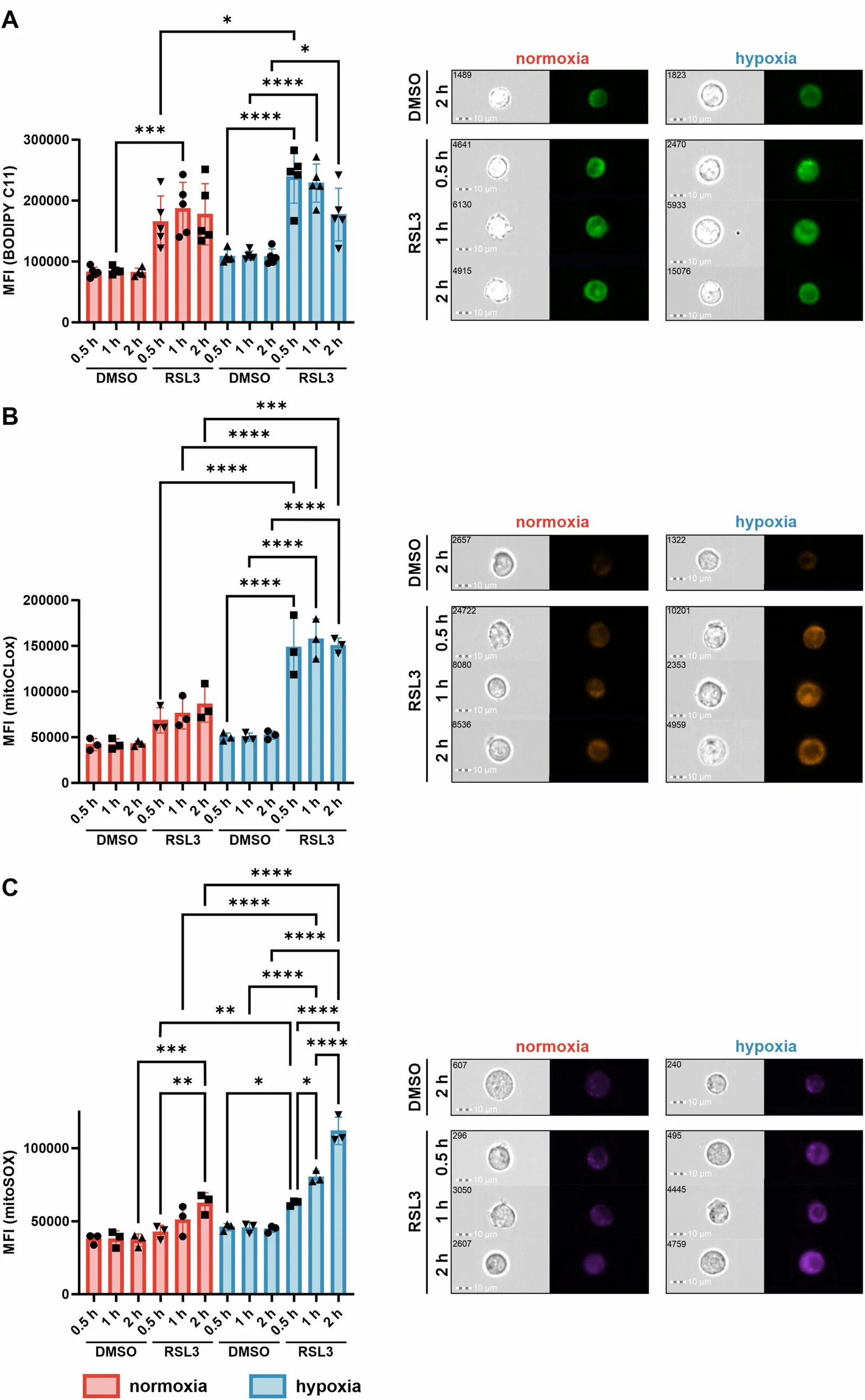
**Chronic hypoxia reinforces lipid peroxidation and mitochondrial ROS production.** THP-1 cells were treated with 5 μM RSL3 and DMSO as vehicle control for indicated times under normoxia or chronic hypoxia. Total lipid peroxidation (A), mitochondrial lipid peroxidation (B) and mitochondrial ROS (C) were measured using imaging flow cytometry. Results are expressed as mean fluorescence intensities (MFI). Pictures show representative cells with the respective staining at 40x magnification in green (BODIPY C11), orange (mitoCLox) and purple (mitoSOX). Data are expressed as mean ± SD. One-way ANOVA with Tukey's multiple comparisons test was used. p ≤ 0.05 was considered significant (*: p ≤ 0.05; **: p ≤ 0.01; ***: p ≤ 0.001; ****: p ≤ 0.0001).

With the following experiments we determined the effect of chronic hypoxia on ROS production and lipid peroxidation as essential components of ferroptosis in THP-1 cells (Fig. 2). Cells were treated with RSL3 for 0.5 h, 1 h and 2 h prior to imaging flow cytometry analysis. We noticed increased and accelerated lipid peroxidation under chronic hypoxia with its maximum after 0.5 h (Fig. 2A). Similarly, mitochondrial lipid peroxidation increased under chronic hypoxia (Fig. 2B). While under normoxia mitochondrial lipid peroxidation steadily increased during 2 h of RSL3, chronic hypoxic cells reached their maximum already after 0.5 h. Mitochondrial ROS increased from 0.5 to 2 h of RSL3 treatment under normoxia as well as chronic hypoxia but reached higher values under prolonged oxygen deprivation (Fig. 2C). All three parameters analyzed indicate a higher sensitivity towards RSL3-induced ferroptosis under chronic hypoxia. While total and mitochondrial lipid peroxidation were fast, mitochondrial ROS progressed more slowly. Control experiments revealed that liproxstatin-1 completely prevented effects of RSL3 on lipid peroxidation and mitochondrial ROS (Fig. S1E and F).

### 3.3 Chronic hypoxia decreases FSP1 to enhance ferroptosis sensitivity

**Fig. 3:**
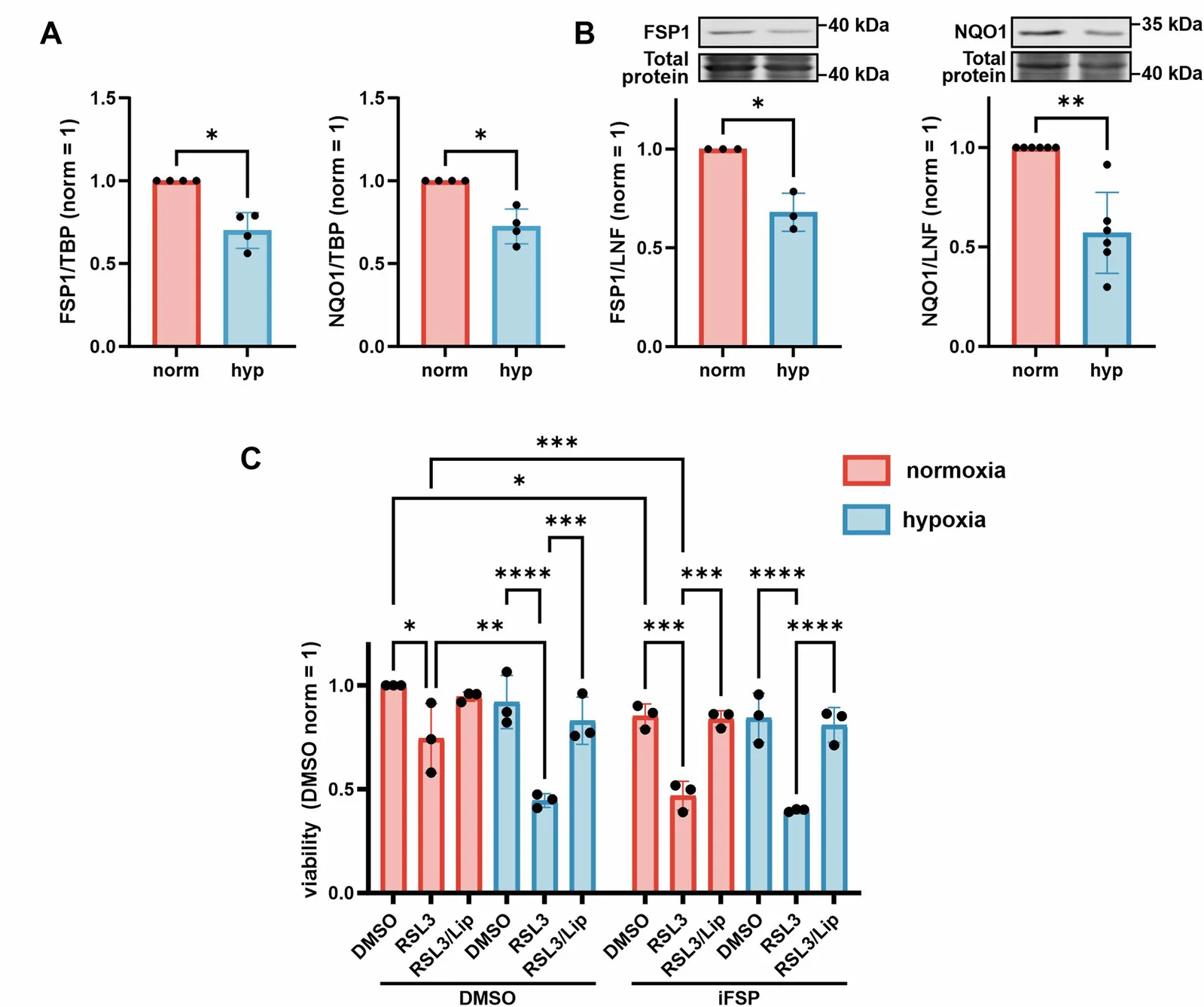
**Chronic hypoxia decreases ferroptosis-associated genes.** (A) RNA expression of ferroptosis suppressor protein 1 (FSP1) and NAD(P)H dehydrogenase [quinone] 1 (NQO1) in normoxic (norm) and chronic hypoxic (hyp) THP-1 cells were analyzed by RT-qPCR and normalized to TATA-box-binding protein (TBP). Normoxia was set to 1. (B) FSP1 and NQO1 protein in THP-1 cells were determined by Western analysis. Data were normalized by lane normalization factor (LNF) and normoxia was set to 1. (C) THP-1 cells were cultured under normoxia and chronic hypoxia and treated with 5 μM RSL3 and/or 10 μM iFSP ± 1 μM liproxstatin-1 (Lip) or DMSO as vehicle control for 6 h. Viability was determined by CellTiter-Blue® Viability Assay. Data are expressed as mean ± SD. Students paired t-tests or two-way ANOVA with Tukey's multiple comparisons tests were used. p ≤ 0.05 was considered significant (*: p ≤ 0.05; **: p ≤ 0.01; ***: p ≤ 0.001; ****: p ≤ 0.0001).

Elevated levels of lipid peroxides and mitochondrial ROS under chronic hypoxia may indicate an unbalanced antioxidant response system. Consequently, we determined mRNA and protein levels of several antioxidative and ferroptosis-associated genes in THP-1 cells. Under chronic hypoxia, mRNA expression of FSP1, NAD(P)H dehydrogenase [quinone] 1 (NQO1), SLC7A11, and glutamate–cysteine ligase catalytic subunit (GCLC) was significantly downregulated (Fig. 3A and Fig. S2A). Decreased FSP1 and NQO1 expressions were also validated at protein level (Fig. 3B). Both FSP1 and SLC7A11 are known to be strongly associated with ferroptosis. As RSL3 inhibits GPX4 to provoke ferroptosis, downregulation of proteins involved in the GPX4 pathway such as SLC7A11 appear less likely to account for increased death under chronic hypoxia. Thus, we further analyzed the role of FSP1 in association with increased ferroptosis under chronic hypoxia.

To explore the role of FSP1 in more detail, we inhibited FSP1 by using iFSP. We co-treated THP-1 cells with RSL3 and/or iFSP for 6 h before determining cell viability (Fig. 3C). Evidently, the combination of iFSP and RSL3 reduced viability under normoxia to a similar extent as RSL3 alone under chronic hypoxia but showed no additional effect in combination with RSL3 under chronic hypoxia. As iFSP itself was not sufficient to induce ferroptosis, neither under normoxia nor chronic hypoxia, we conclude that loss of FSP1 sensitizes cells to ferroptosis under chronic hypoxia likely by increasing the susceptibility to lipid peroxidation. Our data indicate that the decrease of the NRF2 target gene FSP1 might be crucial to sensitize cells towards ferroptosis under chronic hypoxia. However, NRF2 is known to induce several genes involved in the antioxidative response. To estimate the impact of FSP1, we inhibited other ferroptosis-relevant NRF2 targets, such as system X_c_^−^ by erastin, GCLC by BSO, and NQO1 by ES-936 (Fig. S2B) and measured cell viability. Interestingly, only erastin aggravated the effect of RSL3.

### 3.4 Activation of NRF2 abrogates ferroptosis sensitization under chronic hypoxia

**Fig. 4:**
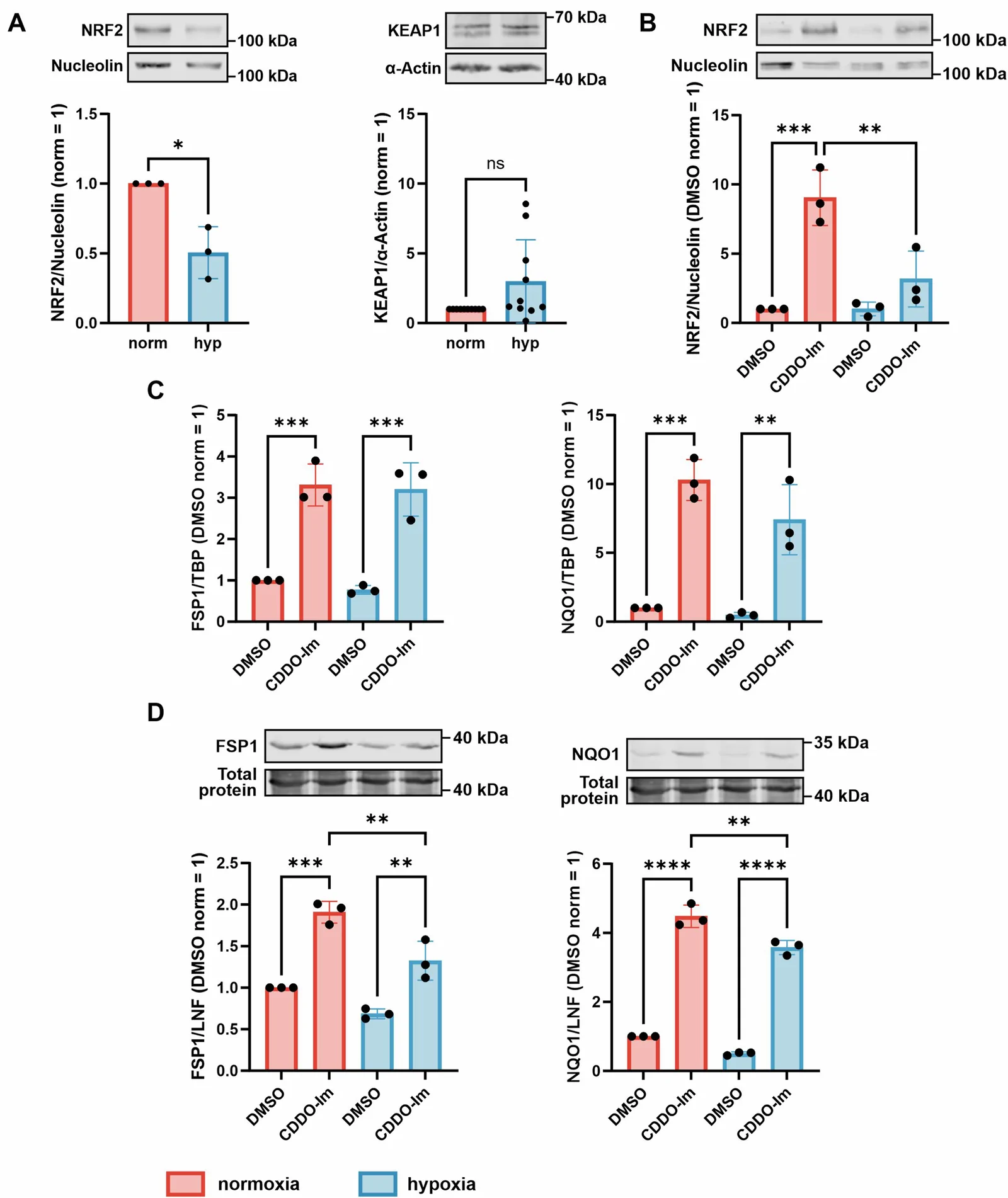
**Chronic hypoxia lowers NRF2 activity.** (A) Nuclear factor erythroid 2-related factor 2 (NRF2) protein amount in nuclear fractions of normoxic (norm) and chronic hypoxic (hyp) THP-1 cells and KEAP1 (Kelch-like ECH-associated protein 1) protein amount in the cytosol were measured using Western analysis. Data were normalized to respective controls (nucleolin for nucleus, α-actin for cytosol). Normoxia was set to 1. (B) Normoxic or chronic hypoxic THP-1 cells were treated with 50 nM CDDO-Im for 24 h before harvest. NRF2 protein amount was analyzed in the nuclear fraction. Data were normalized to nucleolin and the normoxic DMSO control was set to 1. (C) RNA expression of ferroptosis suppressor protein 1 (FSP1) and NAD(P)H dehydrogenase [quinone] 1 (NQO1) were analyzed after treatments with 50 nM CDDO-Im for 24 h by RT-qPCR and normalized to TATA-box-binding protein (TBP). The normoxic DMSO control was set to 1. (D) Protein levels of FSP1 and NQO1 were determined by Western analysis. Data were normalized to the lane normalization factor (LNF) and the normoxic DMSO control was set to 1. Data are expressed as mean ± SD. Students paired t-tests or one-way ANOVA with Tukey's multiple comparisons test were performed. p ≤ 0.05 was considered significant (*: p ≤ 0.05; **: p ≤ 0.01; ***: p ≤ 0.001; ****: p ≤ 0.0001).

Next, we reached out to mechanistically understand decreased expression of the putative NRF2 target FSP1, under chronic hypoxia. To evaluate NRF2 activity, we isolated nuclei from THP-1 cells and analyzed NRF2 abundance under normoxia and chronic hypoxia (Fig. 4A and Fig. S2C). Apparently, chronic hypoxia significantly decreased nuclear NRF2. Considering that translocation of NRF2 into the nucleus is tightly controlled by KEAP1 we measured KEAP1 by Western analysis and observed its upregulation under chronic hypoxia.

To validate the KEAP1-NRF2-FSP1 signaling axis under chronic hypoxia, we used the KEAP1 inhibitor CDDO-Im, which consequently stabilizes and activates NRF2. CDDO-Im directly binds to KEAP1, prevents NRF2 degradation and thereby promotes NRF2 nuclear accumulation [39]. THP-1 cells were treated with CDDO-Im followed by nuclear isolation and revealed increased nuclear abundance of NRF2 (Fig. 4B). Correspondingly, CDDO-Im elevated the NRF2 target genes FSP1, SLC7A11, NQO1, and GCLC at mRNA (Fig. 4C and Fig. S2D) as well as FSP1 and NQO1 at protein level (Fig. 4D). However, activation of NRF2 was weaker under chronic hypoxia compared to normoxia, probably due to decreased translation [40].

**Fig. 5:**
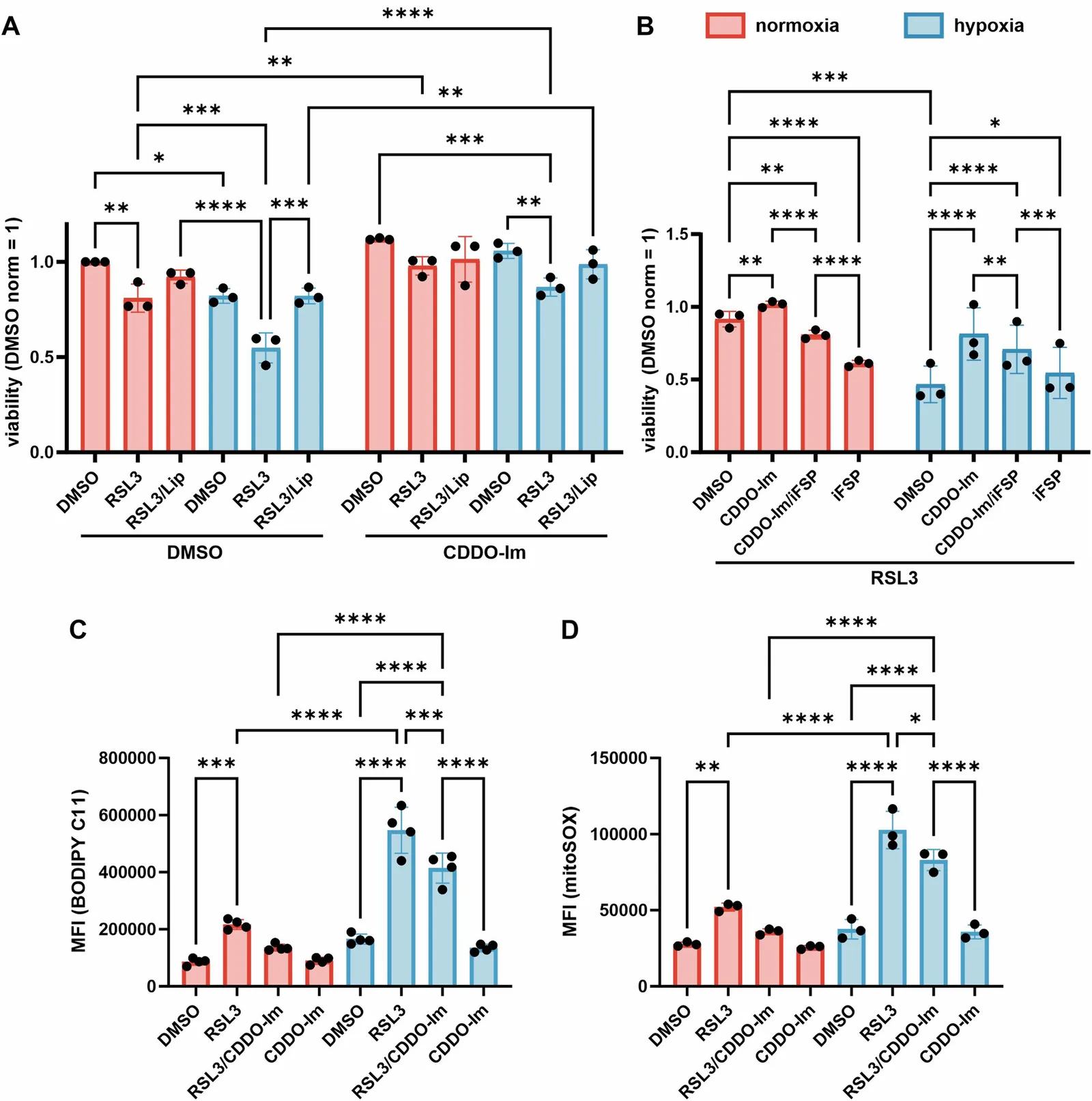
**Activation of NRF2 attenuates ferroptosis sensitization under chronic hypoxia.** (A) THP-1 cells were treated with 25 nM CDDO-Im or DMSO for 24 h and with 5 μM RSL3 ± 1 μM liproxstatin-1 (Lip) or DMSO as vehicle control for 6 h under normoxia vs. chronic hypoxia. Viability was determined by CellTiter-Blue® Viability Assay. Two-way ANOVA with Tukey's multiple comparisons tests were used. (B) THP-1 cells were pretreated with 25 nM CDDO-Im or DMSO for 24 h. Stimulation was with 5 μM RSL3 and/or 10 μM iFSP or DMSO as vehicle control for 6 h under normoxia vs. chronic hypoxia. Viability was determined by CellTiter-Blue® Viability Assay. Two-way ANOVA with Tukey's multiple comparisons tests were performed. (C) THP-1 cells were cultured under normoxia and chronic hypoxia and treated with 50 nM CDDO-Im or DMSO for 24 h and 30 min before analysis with RSL3 and DMSO as vehicle control. Lipid peroxidation was analyzed by imaging flow cytometry and presented as mean fluorescence intensities (MFI). One-way ANOVA with Tukey's multiple comparisons test was used. (D) THP-1 cells were cultured under normoxia and chronic hypoxia and treated with 50 nM CDDO-Im or DMSO for 24 h and 2 h before analysis with RSL3 or DMSO as vehicle control. Mitochondrial ROS were measured using imaging flow cytometry and presented as mean fluorescence intensities (MFI). One-way ANOVA with Tukey's multiple comparisons test was used. All data are expressed as mean ± SD. p ≤ 0.05 was considered significant (*: p ≤ 0.05; **: p ≤ 0.01; ***: p ≤ 0.001; ****: p ≤ 0.0001).

Along these lines, activation of NRF2 via CDDO-Im under chronic hypoxia prevented the ferroptosis-sensitizing effect of RSL3 (Fig. 5A). To estimate the effect of FSP1 induction by CDDO-Im, we combined CDDO-Im with iFSP (Fig. 5B). Inhibition of FSP1 significantly reversed the CDDO-Im mediated rescue upon RSL3 treatment under normoxia and chronic hypoxia, indicating a major contribution of FSP1 on the CDDO-Im protective impact. Fig. S3 shows the complete data set including liproxstatin-1 controls. Additionally, we analyzed lipid peroxidation and mitochondrial ROS in CDDO-Im treated cells. While CDDO-Im significantly reduced these RSL3-induced parameters under chronic hypoxia, it only had a minor impact during normoxia (Fig. 5C and D). Conclusively, decreased NRF2 activity under chronic hypoxia increases ferroptosis susceptibility.

In literature, hypoxia is often associated with activation of NRF2 [41]. Time kinetics starting from acute hypoxia (6 h) to chronic hypoxia (7 days) indeed revealed increased nuclear NRF2 abundance after 6 h of hypoxia (i.e. acute hypoxia) (Fig. S4A). Correspondingly, NRF2 target gene expression of FSP1, SLC7A11, NQO1 and GCLC increased after 6 h of hypoxia (Fig. S4B). However, increased expression of NRF2 target genes declined with prolonged hypoxia, reaching lowest values compared to normoxia at 7 days. Moreover, nuclear NRF2 decreased under prolonged hypoxia. The hypoxic NRF2 regulation was reflected by viability, which showed no response to RSL3 under acute hypoxia where NRF2 was increased but elevated ferroptosis under chronic hypoxia where NRF2 decreased (Fig. S4C). Conclusively, nuclear NRF2 abundance, respectively its activity is differentially regulated during acute versus chronic hypoxia.

### 3.5 Chronic hypoxia decreases FSP1 by reducing NRF2 activity in primary human macrophages

**Fig. 6:**
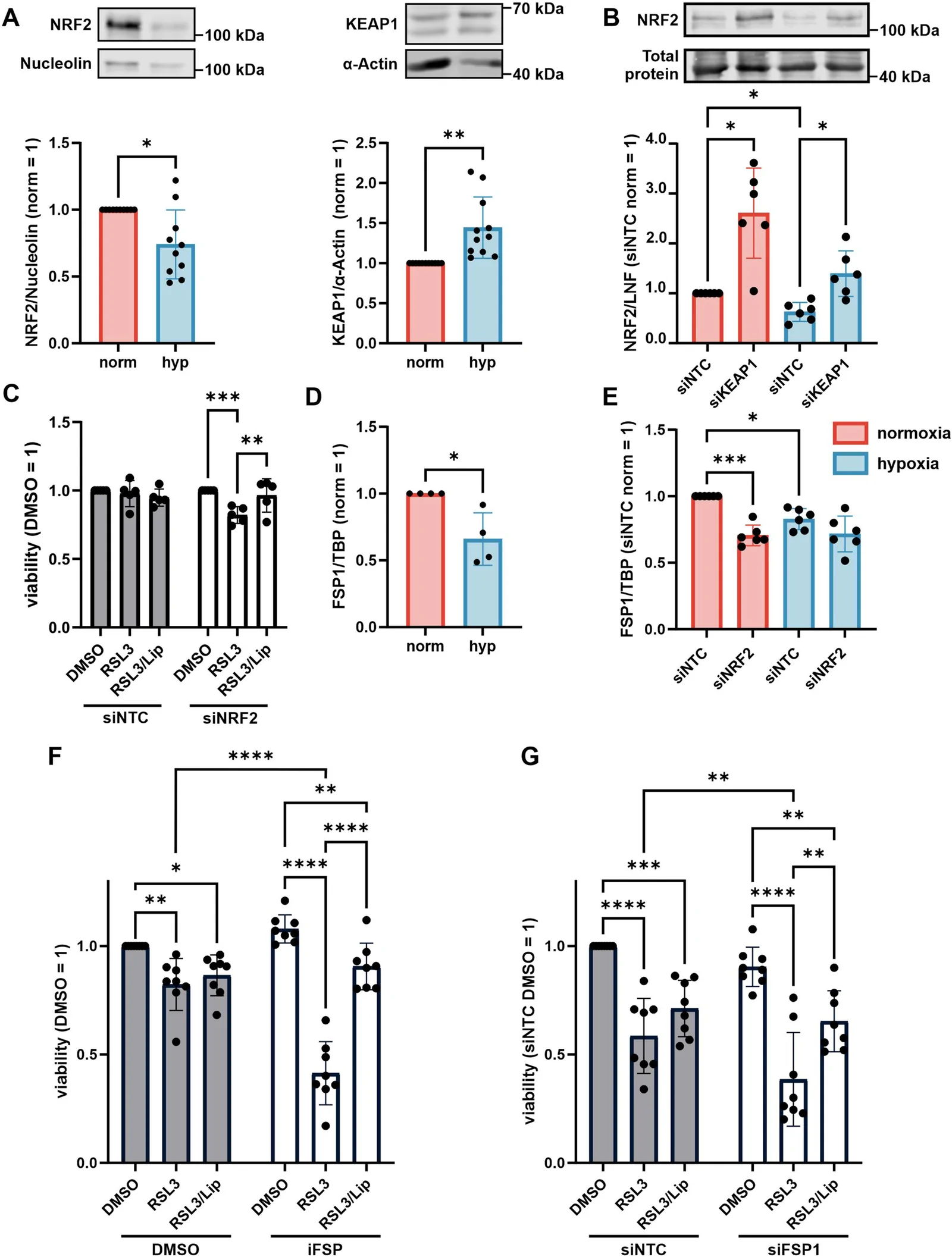
**Chronic hypoxia decreases FSP1 by reducing NRF2 activity in primary human macrophages.** (A) Primary human macrophages were cultured under normoxia and chronic hypoxia. Protein levels and localization of nuclear factor erythroid 2-related factor 2 (NRF2) in the nucleus and Kelch-like ECH-associated protein 1 (KEAP1) in the cytosol were measured using Western analysis. Data were normalized to the respective controls (nucleolin for the nucleus, α-actin for cytosol). Normoxia (norm) was set to 1. Students paired t-tests were performed. (B) Primary human macrophages were transfected with siRNA targeting KEAP1 and afterwards cultured under normoxia and chronic hypoxia. Protein levels of NRF2 were determined by Western analysis. Expression was normalized by lane normalization factor (LNF) and normoxic non-targeted control (NTC) was set to 1. One-way ANOVA with Tukey's multiple comparisons tests were used. (C) Primary human macrophages were transfected with siRNA against NRF2 or a non-targeted control and treated with 5 μM RSL3 ± 1 μM Lip or DMSO as vehicle control for 6 h. Viability was determined by CellTiter-Blue® Viability Assay. DMSO control was set to 1. Two-way ANOVA with Tukey's multiple comparisons tests were used. (D) Primary human macrophages were cultured under normoxia and chronic hypoxia and RNA expression of ferroptosis suppressor protein 1 (FSP1) was analyzed by RT-qPCR and normalized to TATA-box-binding protein (TBP). Normoxia was set to 1. Students paired t-tests were performed. (E) Primary human macrophages were transfected with siRNA targeting NRF2 and afterwards cultured under normoxia and chronic hypoxia. RNA expression of FSP1 was analyzed by RT-qPCR and normalized to TBP. Normoxic NTC was set to 1. One-way ANOVA with Tukey's multiple comparisons tests were used. (F) Primary human macrophages were treated with 5 μM RSL3 and/or 10 μM iFSP ± 1 μM Lip or DMSO as vehicle control for 24 h. Viability was determined by CellTiter-Blue® Viability Assay. Two-way ANOVA with Tukey's multiple comparisons tests were used. (G) siRNA transfected FSP1 knockdown primary human macrophages were treated with 5 μM RSL3 ± 1 μM Lip or DMSO as vehicle control for 24 h. Cells were transfected with siRNA targeting FSP1 or a non-targeted control. Viability was determined by CellTiter-Blue® Viability Assay. Two-way ANOVA with Tukey's multiple comparisons tests were used. All data are expressed as mean ± SD. p ≤ 0.05 was considered significant (*: p ≤ 0.05; **: p ≤ 0.01; ***: p ≤ 0.001; ****: p ≤ 0.0001).

To verify the chronic hypoxia signaling cascade, culminating in a higher RSL3 sensitivity in THP-1 cells, we performed crucial experiments in primary human macrophages. Macrophages were isolated and cultured under hypoxia for 7 days prior to harvesting. We noticed a decreased abundance of NRF2 in the nucleus and a significantly increased amount of KEAP1 in the cytosol under chronic hypoxia (Fig. 6A). To analyze whether KEAP1 is responsible for the NRF2 decrease under chronic hypoxia, we used siRNA against KEAP1 and measured NRF2 abundance (Fig. S5A and B shows validation of KEAP1 knockdown). The knockdown of KEAP1 indeed prevented the decrease of NRF2 under chronic hypoxia (Fig. 6B). To substantiate that a decrease of NRF2 sensitizes human macrophages under chronic hypoxia for ferroptosis, we mimicked the hypoxic phenotype by transfecting normoxic cells with siRNA against NRF2. The knockdown itself reduced viability significantly by nearly 40% (Figure S5C, Figure S5D and E shows validation of NRF2 knockdown). In the following experiments DMSO controls were set to 1 to explore the impact of RSL3 in control transfected and siNRF2 cells (Fig. 6C). As anticipated, the knockdown of NRF2 enhanced ferroptosis in macrophages. However, the decrease in viability was not as pronounced as in other experiments, possibly as a consequence of the substantial loss of viability caused by the NRF2 knockdown. Corroborating THP-1 data, FSP1 mRNA also decreased in primary human macrophages under chronic hypoxia (Fig. 6D). The expression of FSP1 was regulated by NRF2 as it decreased in NRF2 knockdown cells under normoxia, while no changes were seen under chronic hypoxia, because under chronic hypoxia NRF2 was down anyway (Fig. 6E).

To determine the impact of FSP1 on the ferroptosis-sensitizing effects, we treated primary human macrophages with iFSP and measured an increased ferroptosis sensitivity towards RSL3 (Fig. 6F). To confirm inhibitor studies, we knocked down FSP1 expression by siRNA-technology, which was confirmed at RNA and protein level (Fig. S5F and G). A FSP1 knockdown indeed sensitized primary human macrophages to RSL3-induced ferroptosis (Fig. 6G).

Taken together, decreased NRF2 activation under chronic hypoxia diminished FSP1 expression and thus sensitized primary human macrophages as well as THP-1 cells towards ferroptosis.

## 4 Discussion

Sensitivity of primary human macrophages as well as THP-1 cells to RSL3-induced ferroptosis is drastically increased under conditions of chronic hypoxia. THP-1 cells are a human leukemia monocytic cell line, frequently used as a model for human macrophages due to easier cultivation and less donor-to-donor variability.

By time-dependently determining whole cell versus mitochondrial lipid peroxidation and mitochondrial ROS formation, we followed ferroptosis characterizing parameters towards the GPX4-inhibitor RSL3 under normoxia and chronic hypoxia. Interestingly, only GPX4-inhibitors (RSL3 or ML162) induced ferroptosis under chronic hypoxia, while the system X_c_^−^ inhibitor erastin had no effect. This might be a result of decreased SLC7A11 expression under chronic hypoxia, which is a crucial part of system X_c_^−^ and the decrease of SLC7A11 likely already impaired its function. Chronic hypoxia accelerated lipid peroxidation and mitochondrial ROS formation, suggesting that cellular and mitochondrial lipid peroxidation precedes mitochondrial ROS formation. This is in line with the proposed action of RSL3 in accumulating lipid peroxides to provoke ferroptosis. Therefore, we postulate that lipid peroxidation initiates ferroptosis and concomitantly increases mitochondrial ROS. Further, we observed that lipid peroxidation increases faster under chronic hypoxia, likely resulting from a decrease of FSP1, which protects cells from lipid peroxidation. While the importance of lipid peroxidation for ferroptosis is undisputed, the role of mitochondrial ROS remains an open issue. Some reports state that mitochondria and thus, mitochondrial ROS are not necessary for ferroptosis, because cells lacking mitochondrial DNA are still susceptible to ferroptosis even though they have no functional respiratory chain [26]. Others claim that mitochondrial ROS are essential for ferroptosis execution [[42], [43], [44]]. Our study adds to this controversy in showing that lipid peroxidation precedes ROS formation, albeit this does not imply a fundamental role of ROS in ferroptosis execution. Thus, we propose that accelerated and elevated total and mitochondrial lipid peroxidation promotes cell death under chronic hypoxia.

Downregulation of FSP1 under chronic hypoxia increased susceptibility towards ferroptosis. Inhibition or knockdown of FSP1 under normoxia also sensitized THP-1 cells and primary human macrophages towards ferroptosis, thereby mimicking the impact of chronic hypoxia. In addition, we used inhibitors for system Xc-, GCLC, and NQO1, which are known to induce ferroptosis and at the same time are established NRF2 target genes (Figure S2 B). Although inhibition of system Xc-in combination with RSL3 showed a trend to facilitate ferroptosis, none of the inhibitors significantly provoked ferroptosis, highlighting the importance of FSP1 antagonizing this from of cell death. These data point to a crucial role of the NRF2-FSP1 axis for sensitizing cells to ferroptosis under chronic hypoxia. Since activation of NRF2 by CDDO-Im protected from ferroptosis and was not completely antagonized by FSP1 inhibition, we cannot fully rule out that NRF2 target genes, not elucidated in this study, might be involved.

To understand FSP1 regulation under hypoxia, we analyzed the NRF2-pathway. Knockdown of NRF2 reduced the expression of FSP1, thereby validating FSP1 as a NRF2 target gene. Previous studies observed NRF2 activation under hypoxia [41,45,46]. This is corroborated by our data but applies to acute hypoxia, only (Fig. S4). Under chronic hypoxia, NRF2 translocation to the nucleus and its target gene expression were decreased. Mechanistically, we observed increased levels of KEAP1 in the cytosol of THP-1 cells and primary human macrophages under chronic hypoxia. KEAP1 functions as a negative regulator of NRF2 and inhibition of KEAP1 (and thereby activation of NRF2) by CDDO-Im prevented ferroptosis-sensitizing effects under chronic hypoxia [37,39]. In line, our data confirm regulation of NRF2 by KEAP1 under chronic hypoxia. Nevertheless, the question of how KEAP1 increases under chronic hypoxia requires further investigation. Our data highlights the importance of considering severity and length of oxygen deprivation, making NRF2 a potential marker for distinguishing acute from chronic hypoxic macrophages.

This study provides insights how chronic hypoxic regulates ferroptosis in macrophages. In diabetes, hypoxia often occurs in tissues because of damaged blood vessels. This may lead to chronic hypoxia in organs such as the kidney, resulting in diabetic nephropathy which is linked to ferroptosis [47,48]. Thus, activation of NRF2 in chronic hypoxic diabetic nephropathy might be a potential treatment option to inhibit ferroptosis and alleviating organ damage. Alternatively, ferroptosis-sensitizing effects under chronic hypoxia might be beneficial for cancer treatment. Future studies have to show whether decreased NRF2 activity can also be observed in tumors, where hypoxic regions are frequently vascularized provoking intermittent hypoxia and accumulation of tumor promoting macrophages [[49], [50], [51]]. Increasing the sensitivity to ferroptosis may help to overcome therapy resistance, which is often is linked to macrophage accumulation [2,[52], [53], [54]]. Moreover, FSP1 appeared as a promising target for ferroptosis induction in cancer. Recently, two studies showed that inhibition of FSP1 restricted tumor growth, which highlight the potential of targeting FSP1 as a treatment strategy [55,56]. The decrease of FSP1 we observed under chronic hypoxia may enhance the accessibility of cells towards therapeutic ferroptosis induction and influence the dosage of FSP1-inhibting drugs.

## 5 Conclusion

**Fig. 7:**
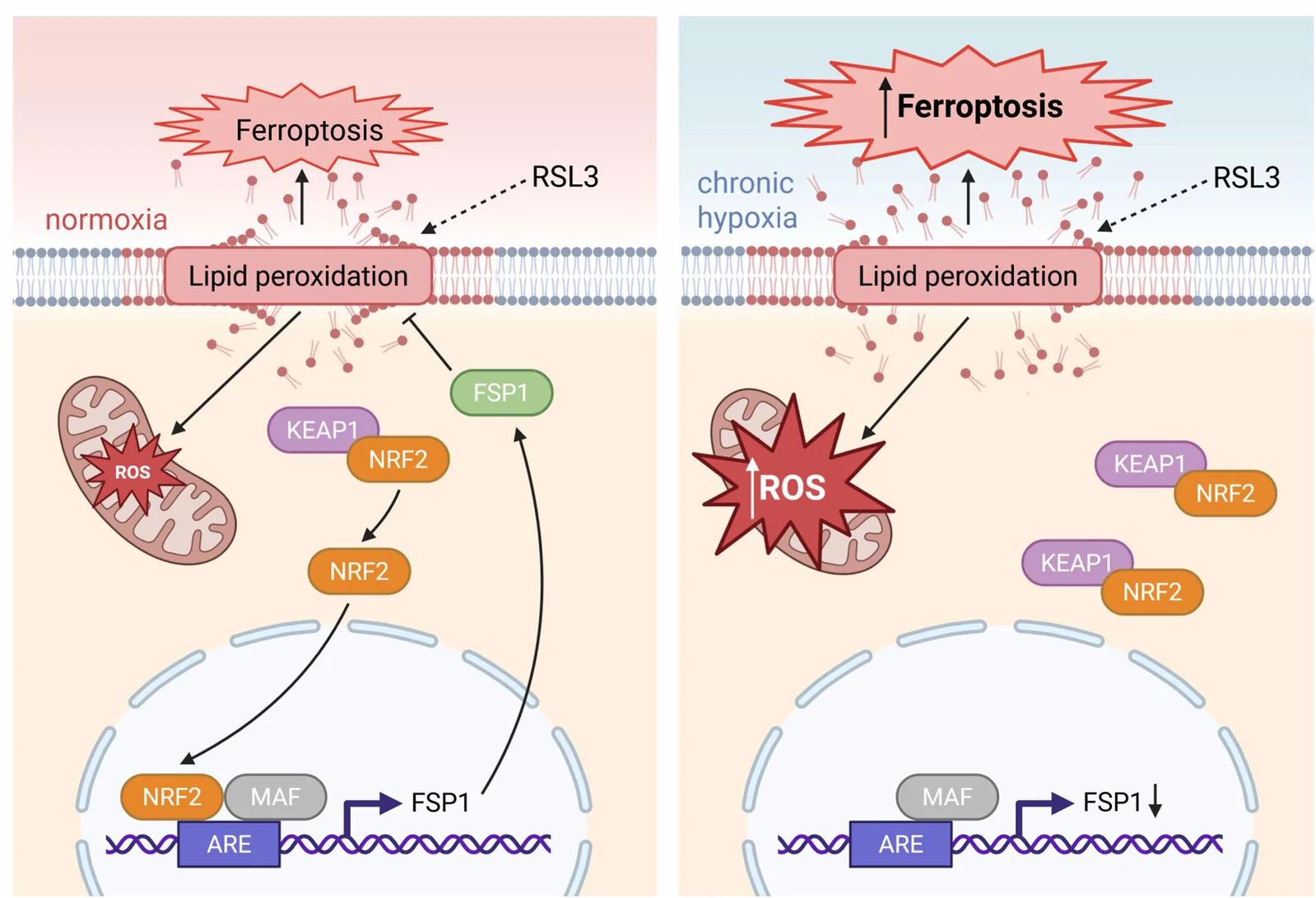
**Graphical summary.** Under normoxia nuclear factor erythroid 2-related factor 2 (NRF2) translocates to the nucleus, forms a heterodimer with small musculoaponeurotic fibrosarcoma proteins (MAF), binds to antioxidant response element (ARE), and induces the expression of ferroptosis suppressor protein 1 (FSP1). FSP1 scavenges lipid peroxides to attenuate RSL3-induced ferroptosis. Under chronic hypoxia, Kelch-like ECH-associated protein 1 (KEAP1) increases to attenuate NRF2 activation, which lowers FSP1 levels. Reduced FSP1 expression favors the generation of lipid peroxides followed by mitochondrial reactive oxygen species (ROS), which aggravate ferroptosis. Figure created in BioRender.

We explored the response of human primary macrophages to RSL3-induced ferroptosis and noticed a profound sensitization under chronic hypoxia, which mechanistically is driven by reduced NRF2 activation and the concomitantly decreased target gene expression of FSP1 (Fig. 7).

## CRediT authorship contribution statement

**Anja Wickert:** Writing – original draft, Investigation, Formal analysis, Data curation. **Anna Schwantes:** Writing – original draft, Resources, Investigation. **Yara Shadid:** Resources, Formal analysis. **Sofie P. Meyer:** Writing – original draft, Resources, Investigation. **Bernhard Brüne:** Writing – review & editing, Funding acquisition. **Dominik C. Fuhrmann:** Writing – original draft, Supervision, Project administration, Conceptualization.

## Funding

This work was supported by the 10.13039/501100001659Deutsche Forschungsgemeinschaft (GRK2336/2 TP6)

We declare no conflict of interest.

## Declaration of competing interest

The authors declare that they have no known competing financial interests or personal relationships that could have appeared to influence the work reported in this paper.

## Data Availability

Data will be made available on request.
