# Supplementary material for: Decreased FSP1 expression under chronic hypoxia sensitizes human macrophages towards ferroptosis
Source: Redox Biol. 2026 Sep 17;97:104406. doi: 10.1016/j.redox.2026.104406 (PMC13602050; doi:10.1016/j.redox.2026.104406)
Supplement: Supplementary file 1 [file mmc1.pdf]

Figure S1

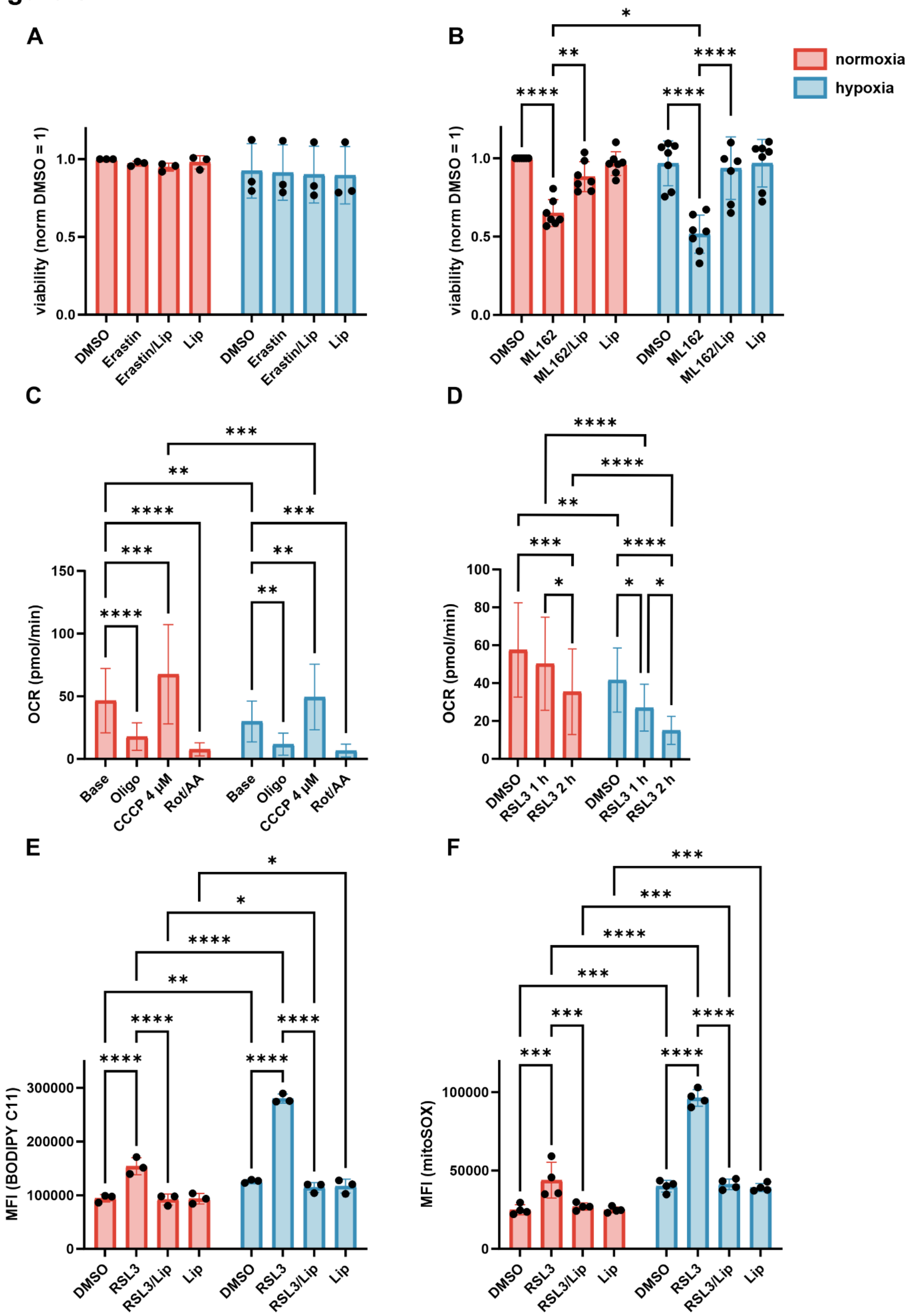
A
B
C
D
E
F
normoxia
hypoxia
viability (norm DMSO = 1)
1.0
0.5
0.0
DMSO
Erastin
Erastin/Lip
Lip
ML162
ML162/Lip
OCR (pmol/min)
150
100
50
0
Base
Oligo
CCCP 4 μM
Rot/AA
80
60
40
20
RSL3 1 h
RSL3 2 h
MFI (BODIPY C11)
300000
200000
100000
RSL3
RSL3/Lip
MFI (mitoSOX)
50000
*
**
***
****

Figure S1: Ferroptosis and mitochondrial integrity under chronic hypoxia. (A) THP-1 cells were cultured under normoxia and chronic hypoxia and treated with 10 µM erastin ± 1 µM liproxstatin-1 (Lip) or DMSO as vehicle control for 6 h. Viability was determined by CellTiter-Blue® Viability Assay. (B) THP-1 cells were cultured under normoxia and chronic hypoxia and treated with 5 µM ML162 ± 1 µM Lip or DMSO as vehicle control for 6 h. Viability was determined by CellTiter-Blue® Viability Assay. (C) The Oxygen consumption rate (OCR) of normoxic and chronic hypoxic THP-1 cells was measured by Seahorse, and mitochondrial integrity was analyzed by sequential addition of oligomycin (2.5 µM), CCCP (4 µM), and rotenone/antimycin A (0.5 µM). (D) Normoxic and chronic hypoxic THP-1 cells were treated with RSL3 (5 µM) for indicated times followed by Seahorse analyses. (E) THP-1 cells were cultured under normoxia and chronic hypoxia and treated with 5 µM RSL3 ± 1 µM Lip or DMSO as vehicle control for 0.5 h. Total lipid peroxidation was stained with BODIPY C11 and measured using imaging flow cytometry. Results are expressed as mean fluorescence intensities (MFI). (F) THP-1 cells were cultured under normoxia and chronic hypoxia and treated with 5 µM RSL3 ± 1 µM Lip or DMSO as vehicle control for 2 h. Mitochondrial ROS were stained with mitoSOX and measured using imaging flow cytometry. Results are expressed as mean fluorescence intensities (MFI). All data are expressed as mean ± SD. Two-way ANOVA with Tukey's multiple comparisons test was performed. $p \leq 0.05$ was considered significant (*: $p \leq 0.05$; **: $p \leq 0.01$; ***: $p \leq 0.001$; ****: $p \leq 0.0001$).

Figure S2

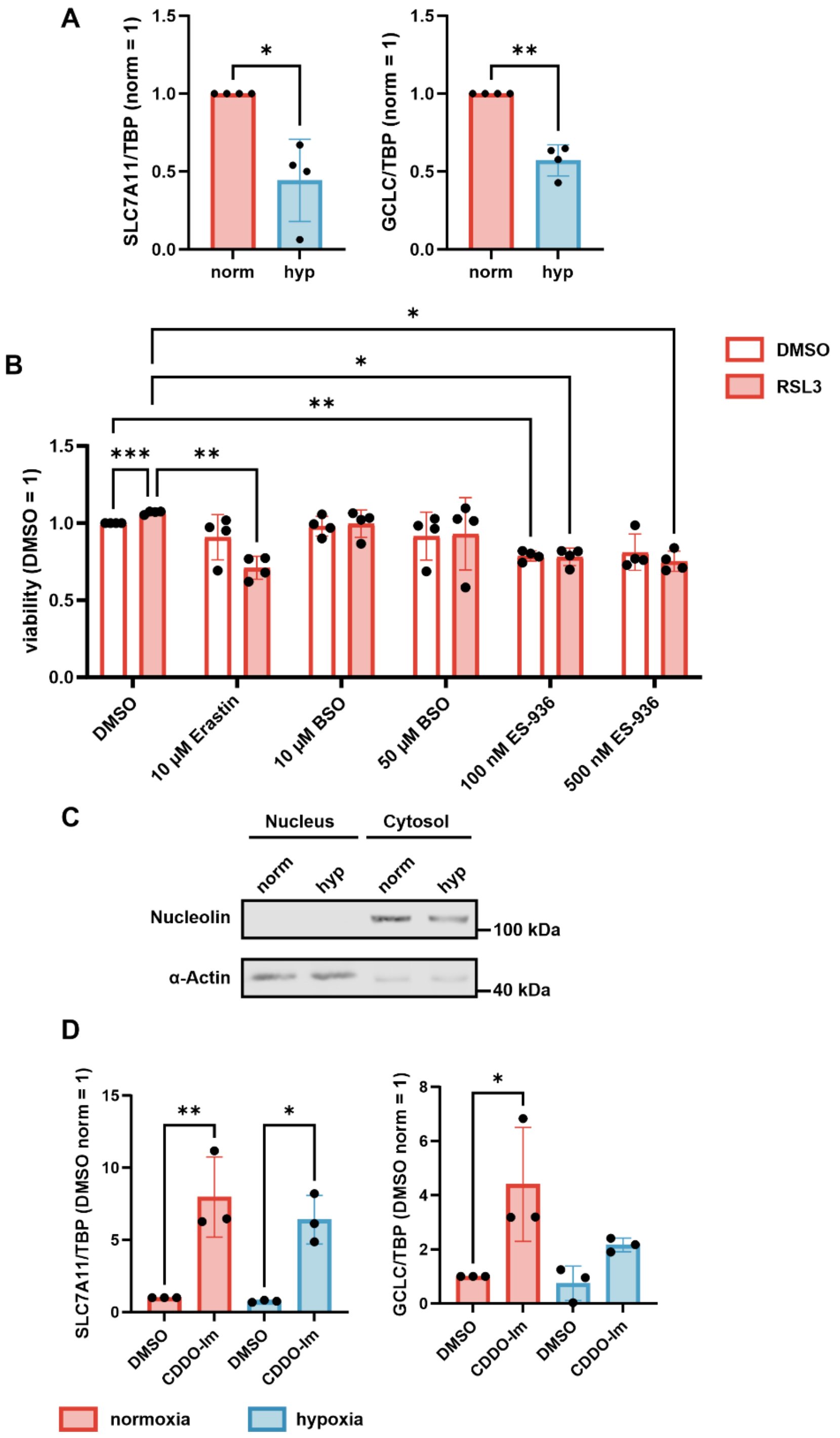

A
SLC7A11/TBP (norm = 1)
1.5
1.0
0.5
0.0
norm
hyp
*
GCLC/TBP (norm = 1)
**
B
DMSO
RSL3
viability (DMSO = 1)
***
**
*
DMSO
10 µM Erastin
10 µM BSO
50 µM BSO
100 nM ES-936
500 nM ES-936
C
Nucleus
Cytosol
norm
hyp
Nucleolin
α-Actin
100 kDa
40 kDa
D
SLC7A11/TBP (DMSO norm = 1)
15
10
5
0
CDDO-Im
GCLC/TBP (DMSO norm = 1)
8
6
4
2
normoxia
hypoxia

Figure S2: NRF2 target genes under chronic hypoxia and CDDO-Im. (A) THP-1 cells were cultured under normoxia and chronic hypoxia. RNA expression of SLC7A11 and glutamate–cysteine ligase catalytic subunit (GCLC) were analyzed by RT-qPCR. Students paired t-tests were performed. (B) THP-1 cells were treated with 5 µM RSL3 in combination with 10 µM erastin, BSO (10 µM or 50 µM), or ES-936 (100 nM or 500 nM). Viability was determined by CellTiter-Blue® Viability Assay. Two-way ANOVA with Tukey's multiple comparisons tests were used. (C) Subcellular fractionation of nuclei versus cytosol. Purity of fractionations was determined by Western analysis using nucleolin for nuclei and α-actin for cytosol. A representative blot of THP-1 cells is shown. (D) THP-1 cells were cultured under normoxia and chronic hypoxia. Cells were treated with 50 nM CDDO-Im for 24 h before harvest. RNA expression of SLC7A11 and glutamate–cysteine ligase catalytic subunit at normoxia vs. chronic hypoxia were analyzed by RT-qPCR. One-way ANOVA with Tukey's multiple comparisons test was performed. RNA expression was normalized to TATA-box-binding protein (TBP). The respective controls were set to 1. All data are expressed as mean ± SD. $p \leq 0.05$ was considered significant (*: $p \leq 0.05$; **: $p \leq 0.01$; ***: $p \leq 0.001$; ****: $p \leq 0.0001$).

Figure S3

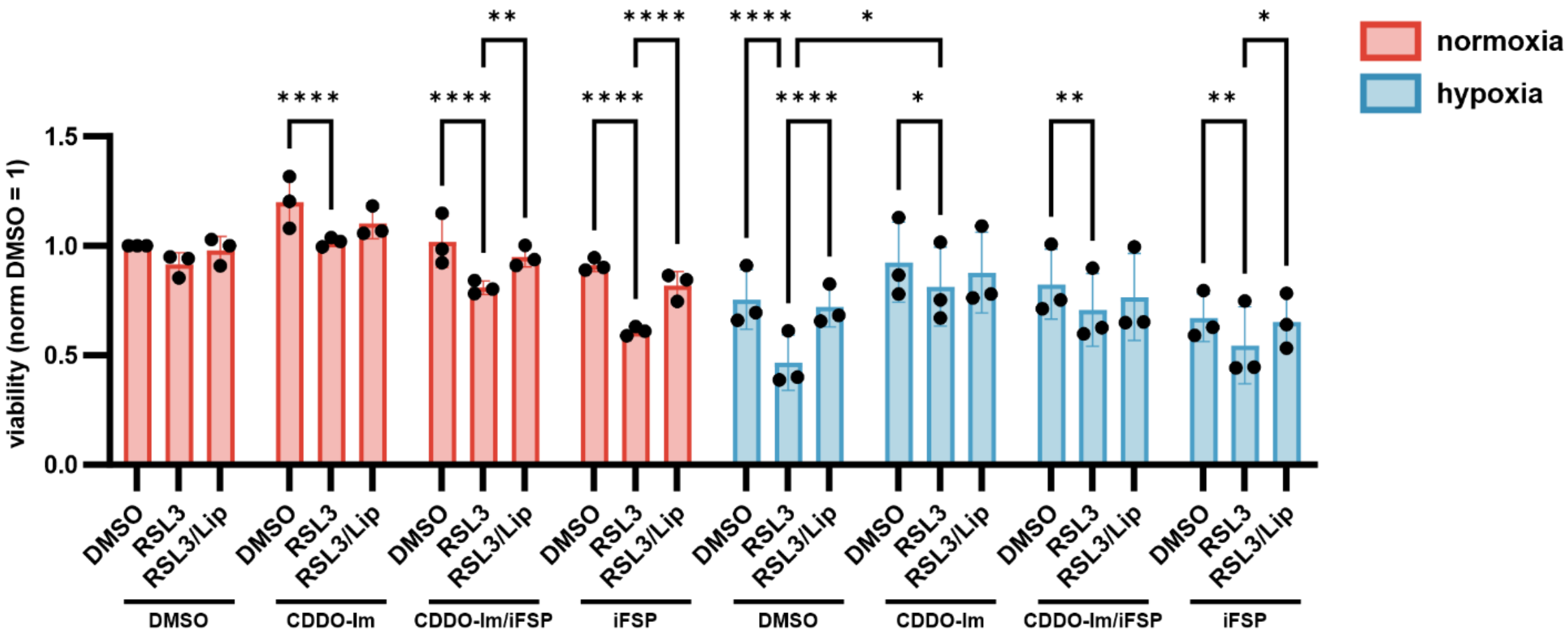

Figure S3: iFSP reduces protective effects of CDDO-Im. Normoxic and chronic hypoxic THP-1 cells were treated with 25 nM CDDO-Im (24 h) and/or 10 µM iFSP (6 h) prior to the addition of 5 µM RSL3 and/or 1 µM liproxstatin-1 (Lip) as indicated. Viability was determined by CellTiter-Blue® Viability Assay. Two-way ANOVA with Tukey's multiple comparisons tests were performed. All data are expressed as mean ± SD. $p \leq 0.05$ was considered significant (*: $p \leq 0.05$; **: $p \leq 0.01$; ***: $p \leq 0.001$; ****: $p \leq 0.0001$).

Figure S4

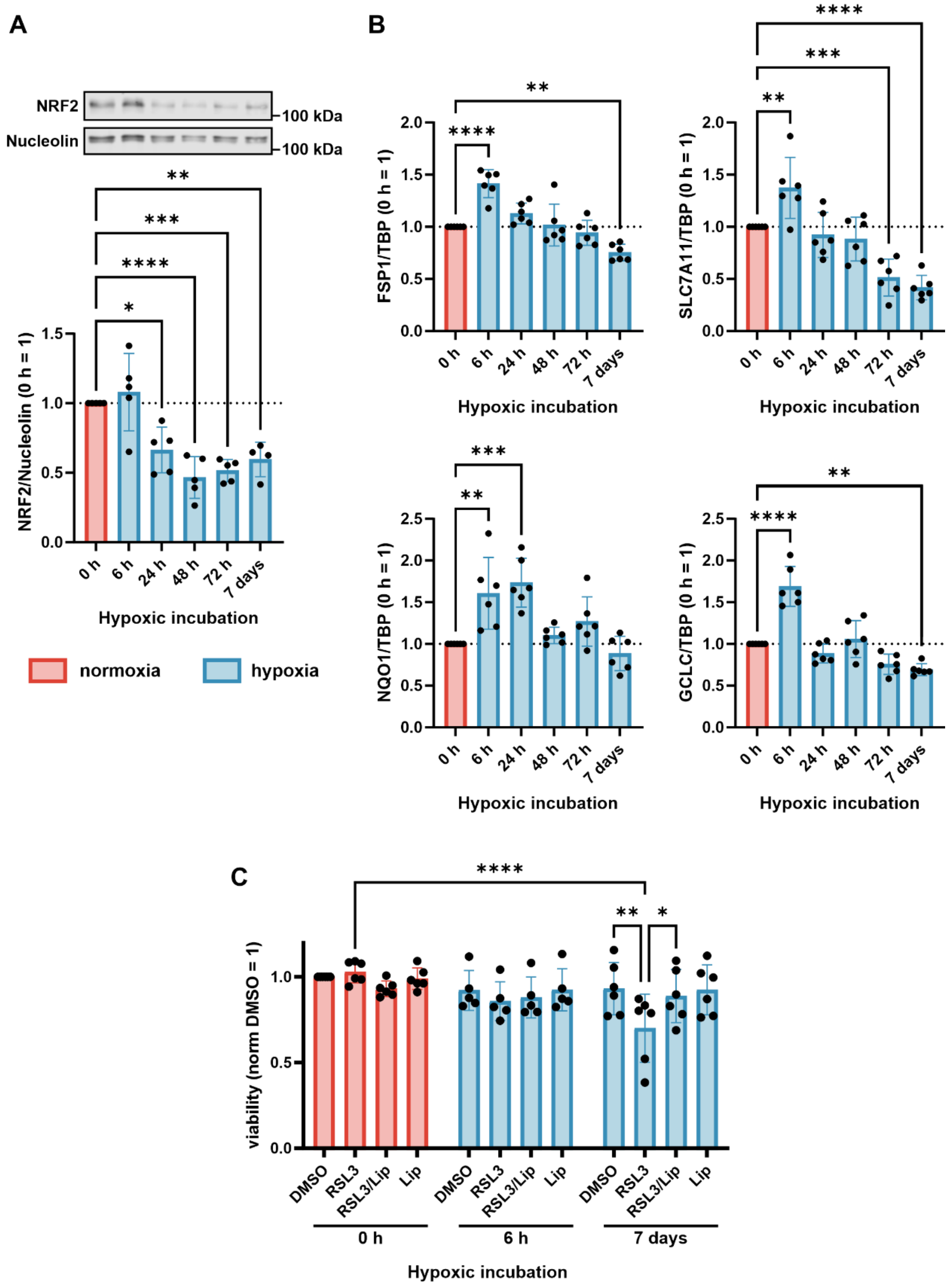

A
NRF2
Nucleolin
100 kDa
100 kDa
NRF2/Nucleolin (0 h = 1)
Hypoxic incubation
0 h
6 h
24 h
48 h
72 h
7 days
normoxia
hypoxia
B
FSP1/TBP (0 h = 1)
SLC7A11/TBP (0 h = 1)
NQO1/TBP (0 h = 1)
GCLC/TBP (0 h = 1)
Hypoxic incubation
C
viability (norm DMSO = 1)
DMSO
RSL3
RSL3/Lip
Lip
0 h
6 h
7 days
Hypoxic incubation

Figure S4: Impact of acute and chronic hypoxia on NRF2 and ferroptosis. THP-1 cells were incubated for indicated times under hypoxia. (A) Nuclear factor erythroid 2-related factor 2 (NRF2) amount in the nucleus measured by Western analysis. Data were normalized to nucleolin at 0 h, set to 1. One-way ANOVA with Dunnett's multiple comparisons test was used. (B) RNA expression of ferroptosis suppressor protein 1 (FSP1), NAD(P)H dehydrogenase [quinone] 1 (NQO1), SLC7A11, and glutamate–cysteine ligase catalytic subunit (GCLC) was analyzed after different time periods under hypoxia by RT-qPCR and normalized to TATA-box-binding protein (TBP) at 0 h, set to 1. One-way ANOVA with Dunnett's multiple comparisons test was used. (C) THP-1 cells were treated with 5 µM RSL3 ± 1 µM liproxstatin-1 (Lip) or DMSO as vehicle control for 6 h under normoxia, acute hypoxia (6 h) or chronic hypoxia (7 days). Viability was determined by CellTiter-Blue® Viability Assay. Two-way ANOVA with Tukey's multiple comparisons tests were performed. All data are expressed as mean ± SD. $p \leq 0.05$ was considered significant (*: $p \leq 0.05$; **: $p \leq 0.01$; ***: $p \leq 0.001$; ****: $p \leq 0.0001$).

Figure S5

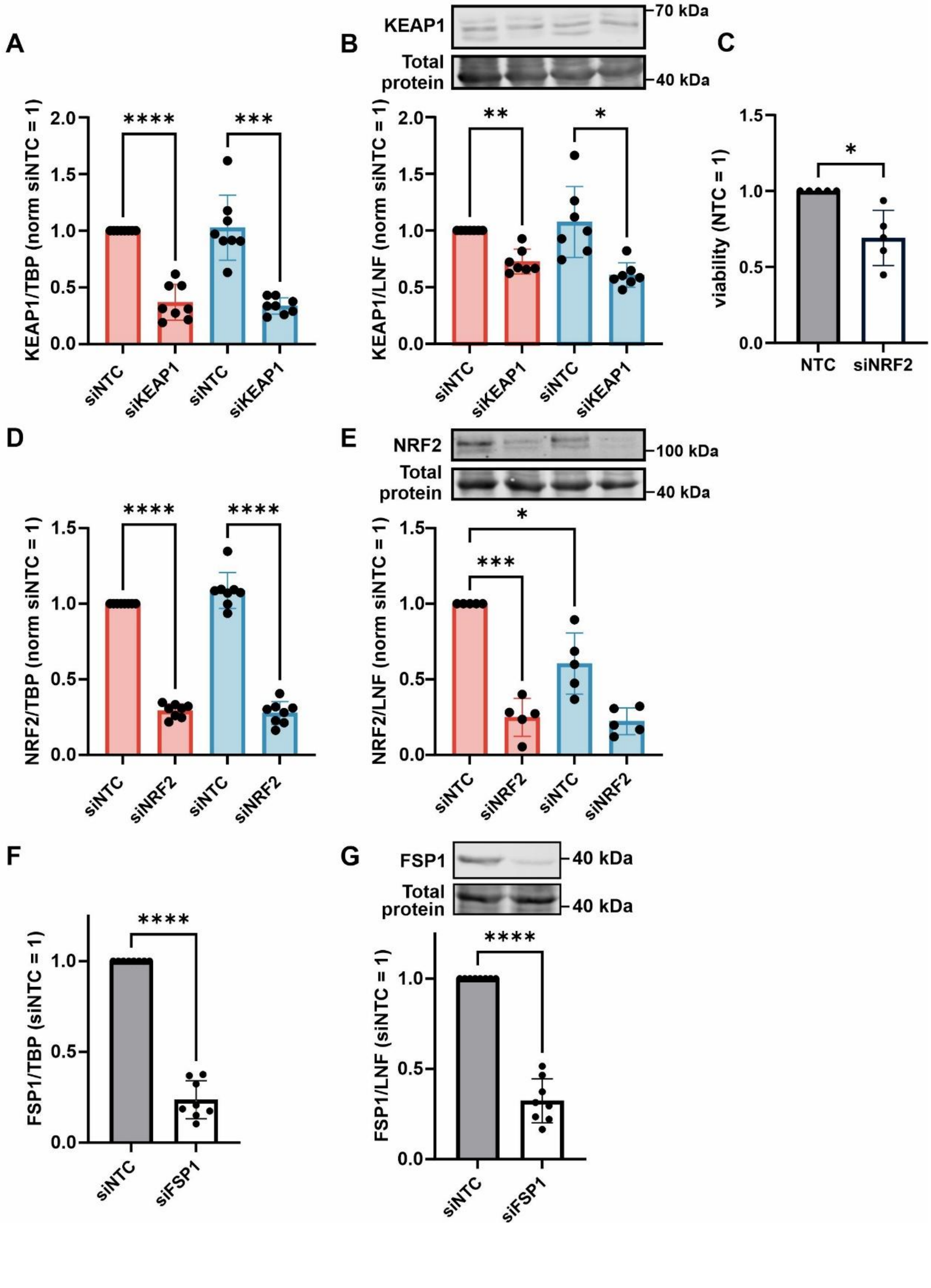

A
B
C
D
E
F
G
KEAP1
Total protein
70 kDa
40 kDa
NRF2
100 kDa
FSP1
KEAP1/TBP (norm siNTC = 1)
KEAP1/LNF (norm siNTC = 1)
viability (NTC = 1)
NRF2/TBP (norm siNTC = 1)
NRF2/LNF (norm siNTC = 1)
FSP1/TBP (siNTC = 1)
FSP1/LNF (siNTC = 1)
siNTC
siKEAP1
siNRF2
siFSP1
NTC
****
***
**
*

Figure S5: Knockdown validation of KEAP1, NRF2, and FSP1. (A - B) Primary human macrophages were transfected with siRNA targeting Kelch-like ECH-associated protein 1 (KEAP1). Knockdown of KEAP1 was validated at (A) mRNA and (B) protein level. mRNA levels were analyzed by RT-qPCR. Proteins were determined by Western analysis. One-way ANOVA with Tukey's multiple comparisons tests were used. (C) Primary human macrophages were transfected with siRNA targeting nuclear factor erythroid 2-related factor 2 (NRF2) and viability was measured by CellTiter-Blue® Viability Assay. Students paired t-tests were performed. (D - E) Primary human macrophages were transfected with siRNA targeting NRF2. Knockdown of NRF2 was validated at (D) mRNA and (E) protein level. mRNA levels were analyzed by RT-qPCR. Proteins were determined by Western analysis. One-way ANOVA with Tukey's multiple comparisons tests were used. (F – G) Primary human macrophages were transfected with siRNA targeting ferroptosis suppressor protein 1 (FSP1). Knockdown of FSP1 was validated at (F) mRNA and (G) protein level. mRNA levels were analyzed by RT-qPCR. Proteins were determined by Western analysis. Students paired t-tests were performed. RNA expression was normalized to TATA-box-binding protein (TBP). Protein expression was normalized by lane normalization factor (LNF). The respective controls were set to 1. All data are expressed as mean ± SD. $p \leq 0.05$ was considered significant (*: $p \leq 0.05$; **: $p \leq 0.01$; ***: $p \leq 0.001$; ****: $p \leq 0.0001$).
